# Telemedical Intervention and Its Effect on Quality of Life in Chronic Heart Failure Patients: The Results from the Telemedicine and e-Health Solution Pilot Program

**DOI:** 10.3390/jcm13092604

**Published:** 2024-04-29

**Authors:** Piotr Wańczura, David Aebisher, Mateusz Wiśniowski, Marek Kos, Hubert Bukowski, Malwina Hołownia-Voloskova, Andrzej Przybylski

**Affiliations:** 1 Department of Cardiology, Medical College of Sciences, University of Rzeszow, 35-310 Rzeszow, Poland; a_przybylski-65@wp.pl; 2 The Ministry of Internal Affairs and Administration Hospital, 35-111 Rzeszow, Poland; m.m.wisniowski@gmail.com; 3 Department of Photomedicine and Physical Chemistry, Medical College, University of Rzeszow, 35-310 Rzeszow, Poland; daebisher@ur.edu.pl; 4 Department of Public Health, Medical University of Lublin, 20-400 Lublin, Poland; marekkos@op.pl; 5 Institute of Innovation and Responsible Development, 02-621 Warsaw, Poland; h.bukowski@innowo.org; 6 Department of Experimental and Clinical Pharmacology, Medical University of Warsaw, 02-097 Warsaw, Poland; malwina.holownia@gmail.com

**Keywords:** telemedicine, quality of life, heart failure, e-health solutions

## Abstract

**(1) Background:** Heart failure (HF) is not only a common cardiovascular disease with a poor prognosis. Its prevalence in developed countries equals 1–2% of the general population of adults, while in Poland HF, patients constitute 3.2% of the total population. Modern heart failure treatment should be focused not only on reducing the risk of death and the number of readmissions due to HF exacerbation but quality of life as well. Telemedicine has been suggested as a viable tool for enhancing HRQL. Therefore, we present the results of telemedical intervention in a group of HF patients and its effect on quality of life in chronic heart failure patients from a pilot study dedicated to reducing social inequalities in health through the use of telemedicine and e-health solutions. **(2) Method:** The project was a multicenter, open, non-controlled trial conducted by the University of Rzeszów, Poland. The data points were collected in the June 2023–December 2023 period from fourteen primary care units from five voivodeships, mostly considered social exclusion areas. A total of 52.7% of the patients recruited were Podkarpackie Voivodeship inhabitants. The result and discussion are presented based on the Chronic Heart Failure Questionnaire (CHFQ) and the EuroQol Visual Analogue Scale (EQVAS). **(3) Results:** During the program, a total of over 100,000 telemedicine examinations were conducted in the form of body weight measurement, heart rate, blood pressure tests, and 7-day Holter or 14-day event Holter assessment. Over the course of this study, coordinating the pilot program medical staff has ordered 570 changes in the patient’s pharmacotherapy, confirming the positive impact on quality of life in the study group. **(4) Conclusions:** A comprehensive telemedical intervention can contribute to an improvement in the quality of life of patients with HF beyond what was achieved with the basic standard of care in the group of HF patients from the social exclusion region. It is now unclear if the result of the basic telemedical intervention would be constant after discontinuation of the mentioned pilot program.

## 1. Introduction

Heart failure (HF) is not only a common cardiovascular disease with a poor prognosis but it is a significant public health problem in different communities worldwide as well [1]. Its prevalence in developed countries equals 1–2% of the general population of adults [2], while in Poland, HF patients constitute 3.2% of the entire population [3]. In addition, heart failure is the most common direct cause of death in Poland, accounting for 9.8% of all deaths [3].

The costs of treating patients with heart failure, especially hospitalizations (90 percent budget allocated for HF treatment), place a heavy burden on the healthcare system [3]. Preventing hospitalization by detecting early evidence of heart failure decompensation in an outpatient setting can improve not only the patient’s quality of life but reduce costs of specialistic care as well [4]. The key situation in optimizing treatment and reducing the number of hospitalizations that worsen the prognosis involves multidisciplinary cooperation between a primary care physician, nurse, and cardiologist. This will not only reduce the risk of death and the number of readmissions due to HF exacerbation but also increase the sense of health by reducing symptoms (e.g., fatigue, dyspnea), improving the quality of life and everyday functioning in the patient’s natural living environment [5]. Multidisciplinary programs are currently the gold standard in the treatment of heart failure. Their possibilities and scope can be significantly expanded using telemedicine, including specific population telemedicine models. Additionally, the COVID-19 pandemic confirmed the effectiveness of telemedicine in relation to different groups of patients [6].

Considering the development of telemedicine, there is no doubt that it improves the quality of healthcare. However, implementing an appropriate telemedicine model requires thorough preparation, defining specific goals, and estimating the spectrum of needs and their cost-effectiveness so that a given telemedicine program does not fail beyond the pilot phase [7].

Telemedicine has been suggested as a viable tool for enhancing HRQL [8]. Timely and effective telemonitoring practices, accompanied by a feedback loop to sufficiently empower patients, has been shown to improve patient’s quality of life [9]. This article aims to confirm the efficacy of telemedicine in improving HRQL in social exclusion areas of Poland by presenting the Polish pilot program ‘Reducing social inequalities in health through the use of telemedicine and e-health solutions’ in the field of cardiology.

## 2. Materials and Methods

### 2.1. Study Design and Population

The goals of this particular project were reducing social inequalities in access to medical care, implementing innovative solutions in the field of telemedicine, shortening the waiting time to obtain specialist medical help, improving the quality of services, ensuring continuity of care, and assessing possible changes in HRQL in the study group. The use of the telemedicine model allows for treatment optimization in the group of recruited heart failure patients by improving cooperation between primary care physicians and cardiologists, increasing the availability of specialist treatment in smaller towns, and carrying out medical interventions aimed at preventing the exacerbation of chronic heart failure. To detect silent AF and significant ventricular arrhythmias, 14-day event monitors or 7-day ECG Holter were used.

**Figure 1:**
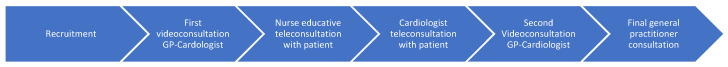
Basic scheme of teleconsultations in the project.

The described project recruited a sample of 429 heart failure patients treated in 5 Polish provinces (voivodeships), mainly from the Podkarpackie Voivodeship (52.7% of the sample). Podkarpackie Voivodeship, localized in the southeast part of Poland, was mainly chosen for the pilot study because of its relatively lower level of industrialization and poorer access to specialistic cardiac ambulatory care compared to more developed regions of Poland. This study recruited heart failure patients treated in primary medical care offices. On the day of recruitment, the cardiologist present at the recruitment site confirmed the presence and advancement of heart failure. For a period of ca. 3 months, these patients were monitored daily in real time (via the GMS network) in terms of body weight and blood pressure using an electronic scale and an electronic blood pressure monitor (measurements 2 times a day plus each time on request). In the event of abnormal weight gain or high or low blood pressure values, defined as atypical compared to the baseline, telephone intervention often combined with pharmacological modification was performed through a selected monitoring center (paramedic/specialist physician) directly after any abnormality detection. Telemonitoring was also accompanied by a series of video or teleconsultations [Figure 1]. Some of the patients (about 20%), mainly with sinus rhythm, were additionally monitored by 7-day Holter or 14-day event Holter due to silent atrial fibrillation or ventricular arrhythmia detection. The Chronic Heart Failure Questionnaire (CHFQ) [9] and EuroQol Visual Analogue Scale (EQVAS) [10], administered at the beginning of the project and after approximately 3 months of participating in the program, were used to quantify HRQL effects of the telemedical intervention. Final-year medical students at the University of Rzeszow supervised the correct completion of the survey forms.

The project was an open, non-controlled trial conducted by the University of Rzeszów, Poland. The data points were collected in the June 2023–December 2023 period and were recruited from 14 primary care units from 5 voivodeships, mostly considered social exclusion areas.

During the program, a total of over 100,000 telemedicine examinations were conducted in the form of body weight measurement, heart rate, blood pressure tests, and 7-day Holter or 14-day event Holter assessment. Over the course of this study, coordinating medical staff ordered 570 changes in the pharmacotherapy of the patients.

### 2.2. Resulting Study Pool

The research material is a group of 346 patients from the total sample. These were the patients who filled out the Polish versions of the CHFQ and EQ-5D-5L questionnaires at the point of inclusion to the program (recruitment) and at the end of it (final general practitioner consultation). This sample includes patients who answered at least one question from the initial and final questionnaire. However, the missing data points in the sample constitute only 4.1% of all data points.

### 2.3. Study Variables

This analysis utilizes total CHFQ results as well as EQVAS results from the EQ-5D-5L questionnaire. Remaining data from the EQ-5D-5L questionnaire have been used to calculate the cost utility of the telemedical intervention. It has been shown that the difference between mean health state utility values (HSUVs) calculated for each patient between the point of final general practitioner consultations and the recruitment reached 0.070 (95% CI 0.050 to 0.090) and was statistically significant at a *p*-value of < 0.0001 using a paired *t*-test [11]. It is, therefore, unnecessary to repeat the same analysis in the same study group.

The CHFQ questionnaire measures disease-specific health-related quality of life and contains information on the use of this test for patients with heart failure. The CHFQ has variations, but for this study specifically, the entire test/measure was used, and no modified versions were considered. There are 16 items with scales of 1 to 7 assessing emotional and physical symptoms of heart failure. Numbers indicating the chosen answer to the specific question were used to calculate the sum constituting three HRQL subscales for dyspnea (5 items), fatigue (4 items), and emotional function (7 items). Higher subscale results represent better HRQL. The EQVAS records the patient’s self-rated health on a vertical visual analog scale where the endpoints are labeled ‘The best health you can image’ and ‘The worst health you can image’. It was used as a quantitative measure of health outcomes that reflects the patient’s own judgment.

### 2.4. Statistical Analysis

Baseline data were summarized using descriptive statistics, such as means and standard deviation (sd) for continuous data with normal distribution, as well as absolute numbers and percentages for categorical data. Comparative analysis between before and after enrolment in the program was performed using a paired *t*-test. Two-sided *p* < 0.05 was considered significant. All analyses were performed using data analysis and statistical software (StatsDirect version 2.8.0).

### 2.5. Ethical Considerations

Consent from the Ethics Committee at the University of Rzeszow number 2023/06/0032 was obtained on 21 June 2023. Patients who met the entry criteria were informed by the researcher about the purposes of this study and participated only after they had given written consent. Participation in this study was on a voluntary basis, and anonymity was preserved. Furthermore, all participants were informed of their rights to refuse or discontinue their participation.

## 3. Results

### 3.1. Descriptive Characteristics

The study pool consisted of 346 patients. Baseline characteristics at the point of inclusion to the project are presented in Table 1. The mean age was 69.8 years (10.3), while the median was 71 years. There was a higher male predominance (60.1%). More than half of the study pool (61.8%) was categorized as II NYHA class. Hypertension was the most prevalent disease (75.4%), followed by dyslipidemia (56.6%). Around one-third of the study pool had a myocardial infarction history (30.3%). One-fourth underwent an angioplasty procedure (24.6%). The most frequently used medication was beta-blockers (84.4%), followed by angiotensin-converting enzyme inhibitors (62.1%), loop diuretics (46.5%), oral anticoagulants (38.4%), and thiazide diuretics (35.0%). Other drugs were used by less than one-fourth of the studied pool. Over the course of this study, coordinating medical staff ordered 570 changes in the patients’ pharmacotherapy in the study group.

### 3.2. HRQL Changes

The average length between the initial and final questionnaire being filled out was 117 days, which is in line with the 3 months adopted in the project planning.

The telemedical intervention seemed to improve HRQL [Table 2]. The changes in all but one measure of HRQL have been positive and statistically significant using a paired *t*-test at a *p*-value of < 0.001. This concerns the dyspnea subscale, which has shown a mean change of 1.41 points (4.7% improvement against the initial mean score), the emotional function subscale with a mean change of 1.01 points (6.0% improvement), and the subjective health state, measured using the EQVAS, with a change of 5.02 (8.6%). Only the fatigue subscale has not been affected by the telemedical intervention.

**Figure 2:**
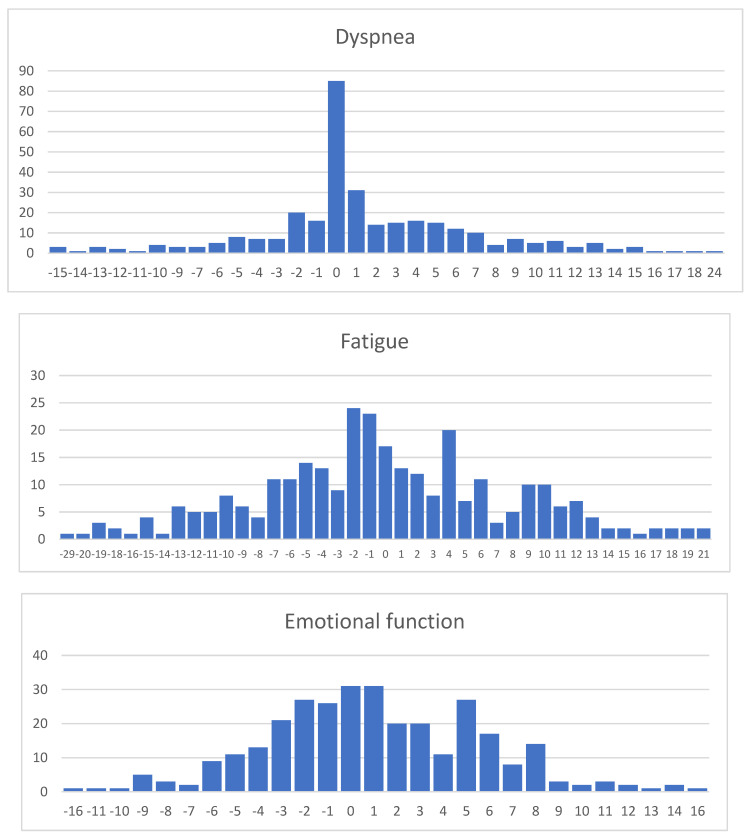

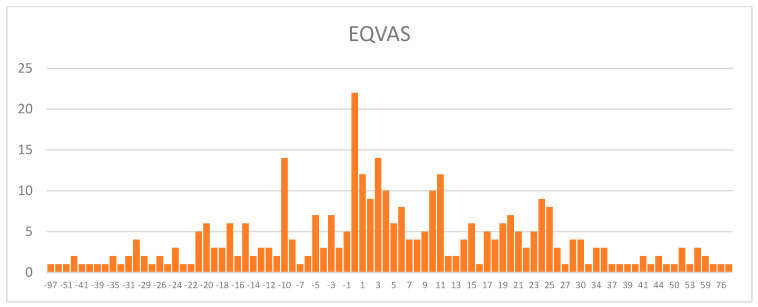
Histograms of changes in dyspnea, fatigue, emotional function, and EQVAS subscales in the study sample.

The changes in the particular patients’ dyspnea, fatigue, emotional function, and EQVAS subscales are presented in Figure 2. A notable feature of these data is that every subscale except for the statistically insignificant fatigue subscale has a median change of 0 points. Thus, the most frequent patients have not seen a positive or a negative change in their HRQL, while the median score (−2 points) indicates that the most frequent patients have experienced a deterioration in the fatigue subscale.

A multivariate analysis of statistically significant subscale changes was conducted with a number of explanatory variables [Table 3]. The number of telemedical examinations and the number of pharmacotherapy modifications were used as indicators of the intensiveness of telemedical intervention. Sex and age were used as indicators of the basic characteristics of the study pool. NYHA class indicates the severity of the disease.

The telemedical intervention was found to have a greater effect on dyspnea condition improvement for female patients, as well as those in higher NYHA classes. Greater emotional function progress concerned patients in higher NYHA classes as well. However, the subjective health state improvement is not dependent on the NYHA class. One confounding result of the analysis was the decrease in EQVAS with a higher number of pharmacotherapeutic modifications.

Overall, the analysis has not found a correlation between the intensiveness of telemedical intervention and HRQL. The HRQL improvement reported in this study was not dependent on age either, while certain HRQL subscales were dependent on NYHA class, with higher severity of the disease connected with a more substantial improvement of HRQL.

**Figure 3:**
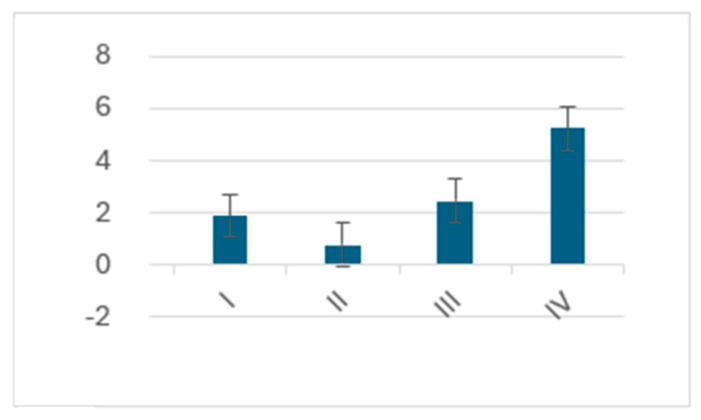
Average dyspnea score change grouped according to NYHA class.

**Figure 4:**
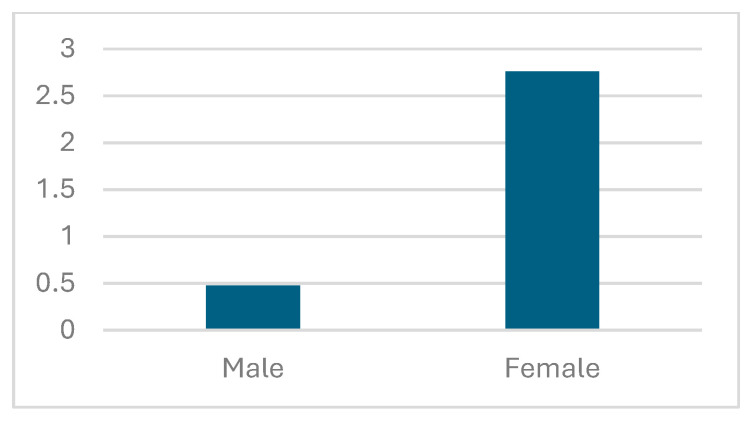
Average dyspnea score change grouped according to sex.

Average dyspnea score improvement was the highest among the IV NYHA class patients, with a ca. 5.2 point increase after the telemedical intervention [Figure 3]. The improvement grew with each stage of the initial NYHA class, except for the I NYHA class patients, who have seen dyspnea score growth higher than for the II NYHA class group. Improvement in the dyspnea score for females equaled ca. 2.8 points and was notably higher than for males, for which a growth of 0.5 points was recorded [Figure 4].

**Figure 5:**
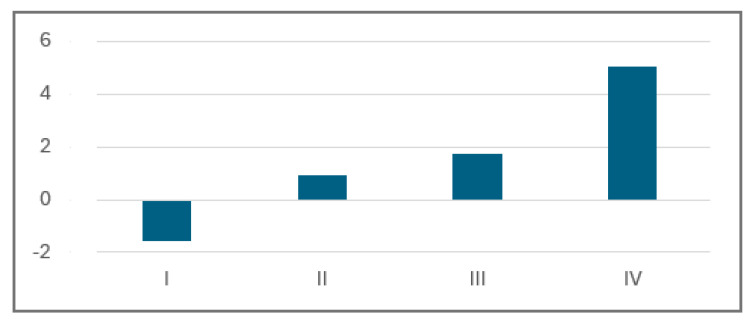
Average emotional function score change grouped according to NYHA class.

There was a visible increase in the average emotional function score for patients with more severe initial heart failure conditions described by the NYHA score. The change in emotional function score varied from a fall of ca. 1.5 points for I NYHA class patients to a ca. 5.1 point improvement for the IV NYHA class group [Figure 5].

**Figure 6:**
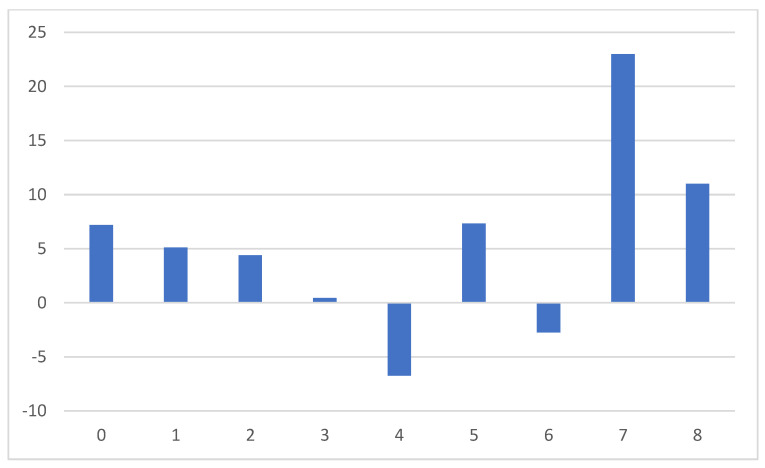
Average EQVAS change grouped according to the number of pharmacotherapy modifications.

Although the statistically significant negative dependence of EQVAS score change on the number of pharmacotherapy modifications was documented, the average changes for patient groups classified on this basis were not coherent. The low and very high number of pharmaceutical modification groups experienced positive EQVAS changes, while patient groups with the number of pharmaceutical modifications in the range of 3 to 6 have seen both negative and positive changes [Figure 6]. This only confirms the need for an individual approach in anticipation of improving the quality of life in heart failure patients. This is the expected therapeutic result of the treatment used, not the number of drugs that are responsible for the change in the level of quality of life.

## 4. Discussion

High prevalence and mortality are only some of the considerable characteristics of heart failure. It is also connected with health-related quality of life (HRQL) impairment [12]. In the presented study, a positive impact of a telemedicine intervention on heart failure patients was achieved. The changes in all but one measure of HRQL have been positive and statistically significant. This concerns dyspnea, which has shown a mean change of 1.41 points (4.7% improvement against the initial mean score), emotional function, with a mean change of 1.01 points (6.0% improvement), and subjective health state measured using EQVAS, with a change of 5.02 (8.6%). Only the fatigue subscale has not been affected by the telemedical intervention in the observed period. The telemedical intervention was found to have a greater effect on dyspnea condition improvement in the female group and patients in a higher NYHA classification. There was a visible increase in the average emotional function score for patients with more severe initial heart failure conditions described by the NYHA score. One confounding result of the analysis was the decrease in EQVAS with a higher number of pharmacotherapeutic modifications, which confirms the need to individualize the approach of every patient’s treatment within the telemedicine model.

Dyspnea, chest pain, sleep disorders, fatigue, and depression are only some of the physical and psychological symptoms of HF. At the same time, quality of life in HF is an extremely crucial element of the course of the disease and treatment process [13]. Although, in most cases, it is a progressive disease, when determining a poor prognosis, we must take into account that poor quality of life is not inevitable, and various medical interventions, including the use of telemedicine solutions, can help maintain or improve the quality of life of patients with heart failure [14].

Improving the methods of monitoring patients, as well as the possibility of optimizing pharmacotherapy through the use of telemedicine solutions, has improved the previously used care strategies for patients with heart failure. We observed the greatest increase in the use of telemedicine solutions during the COVID-19 pandemic, and with each subsequent year, we observed an increase in the importance of telemedicine as an integral part of the hybrid system of care for patients with heart failure [15]. The effect of telemedical intervention in HF patients on their HRQL found in this study corroborates the results seen in the literature. According to Knox et al. [5], who conducted a meta-analysis, when compared to usual care, telemedicine was associated with a small significant increase in overall HRQL. Telemedicine delivered over a duration of over a year and via telemonitoring was the most beneficial. Ten telemedicine studies reported slight improvements in measures of HRQL for HF adults receiving telemedicine compared to those receiving conventional healthcare. Two studies found [16,17] that patients randomly allocated to the remote telemedical management group showed an improved score for physical functioning over the entire study period and quality of life compared with the conventional healthcare group. Ewa et al. [18] reported a statistically significant improvement in HRQL over 3 months of follow-up. GESICA [19] found that the telemedical intervention group had a significantly better quality of life than the control group. The addition of telemedicine to a standard HF program has been found to be an effective measure in improving HRQL in high-risk patients with chronic HF regardless of cognitive function, affective status, or frailty [20].

However, another meta-analysis has not found significant HRQL improvement effects of telemedicine [21]. Five cited studies reported notable but not statistically significant improvements in HRQL before and after the intervention of telemedicine.

One study underlines the importance of QoL in patients with HF [22]. Previous research on patients’ preferences shows that patients give equal or more importance to QoL when compared with length, and about half of the HF patient population is willing to select therapies that improve their QoL, even if it leads to a shortening of life [23,24]; although, one study showed the opposite results [25].

HRQL is a strong and independent predictor of all-cause mortality and HF hospitalization across all geographic regions in mildly and severely symptomatic HF and among patients with preserved and reduced ejection fraction [8]. Quality of life is independently related to survival in a cohort of hospitalized patients with HF [26].

A key factor of this study is the sustainability of improved HRQL. This study seeks to provide a follow-up analysis consisting of additional CHFQ and EQ-5D-5L questionnaires being filled out by the study pool patients after 12 months from recruitment. This is the standard approach in such evaluations. Once a telemedical intervention along with telemonitoring and frequent medical personnel consultation ceases, the improved patient empowerment to manage their disease, adherence to the pharmacotherapy, etc., achieved during the main project might have no impact on outcomes during the extended follow-up [27]. Extending the follow-up period seems crucial, especially since some studies have shown that extending the telemonitoring period to 12 months or more may have a positive effect on overall HRQOL. However, by extending this time beyond 18 months, the positive impact of telemedicine on the physical dimension can be demonstrated [19]. The result of this study confirms the usability of the selected telemedicine model in quality of life improvement. As we know from previous data in the heart failure patients group, not only the length of life but its quality remains important, and the risk of hospitalization due to heart failure exacerbation remains the most crucial factor [28]. The obtained results in the presented pilot study in a population of heart failure patients from social exclusion regions confirm the usability of a very simple telemedicine model and its impact on quality of life in a relatively short period of the study observation. Taking into account the number of people with heart failure in Europe and the expected growth in this field within the next 10 years, the future telemedicine models will be very important in the distribution of access to specialistic cardiac ambulatory care and redirecting some of the patients to primary medical care, especially in non-big city areas. An insufficient percentage of the use of basic groups of drugs with documented impacts on cardiovascular mortality or recurrence of hospitalization was observed in our pilot program. It remains one of the most important reasons for the risk of readmission due to heart failure exacerbation and it, in turn, affects the patient’s quality of life. Regular daily measurements and the awareness, that someone from medical staff in real time will check and evaluate the obtained results may probably increase taking medication and following non-medical recommendations. It not only gives a sense of security but responds positively to the patient’s quality of life as well. The telemedicine models may, in the future, become a basic tool and routine procedure in the heart failure population, influencing medical treatment and accompanying management.

## 5. Limitations

The analysis compared the same group of HF patients for a comparable period before and after inclusion in the project, as there was no control group. This causes a time inconsistency between the comparator and benchmark groups. The lack of use of the control group in the analysis can result in the inertial change in HRQL as the quality of life varies with age. However, the exact direction of age-related change in HRQL for HF patients is not clear. Some studies claim stable HRQL scores with aging [29], while others report improvement after one year [30]. Nevertheless, the effect of aging on HRQL should not be as profound as the change that was observed in only a 3-month period.

Additionally, the time inconsistency can result in the seasonal effects of quality-of-life measures [31]. Typically, these measures rise during spring and summer and decrease in autumn and spring. However, the final scores were obtained in the June–December period, while the initial scores were analyzed in the March–September period. This should work contrary to the observed outcomes.

Thirdly, we did not investigate various factors, such as comorbidities, sleep quality, and the time of heart failure diagnosis, which may be related to the quality of life in heart failure patients.

## 6. Conclusions

Based on the results, it is concluded that a comprehensive telemedical intervention can contribute to improvement in the quality of life of patients with HF beyond what was achieved with a basic standard of care. It is now unclear if the result of the basic telemedical intervention would be constant after the discontinuation of the mentioned program addressed heart failure patients from the social exclusion region in the southeast part of Poland. The scheduled comparative analysis 12 months after finishing the pilot study based on the CHFQ and EQ-5D-5L questionnaires may answer the question.

## Figures and Tables

**Table 1:** Study pool baseline characteristics.

	n = 346
Age, mean (sd)	69.8 (10.3)
Female gender, n (%)	138 (39.9%)
Body mass in kg, mean (sd)	85.2 (16.0)
DBP in mmHG, mean (sd)	70.9 (10.9)
SBP in mmHG, mean (sd)	133.5 (22.2)
HR, mean (sd)	78.5 (12.2)
NYHA class, n (%)	
I	27 (7.8%)
II	214 (61.8%)
IIII	87 (25.2%)
IV	18 (5.2%)
Etiology, n (%)	
Hypertension	261 (75.4%)
Dyslipidaemia	196 (56.6%)
Diabetes	105 (30.3%)
Myocardial infarction history	103 (29.8%)
Atrial fibrillation	100 (28.9%)
Nicotinism	36 (10.4%)
Hypothyroidism	35 (10.1%)
Chronic kidney disease	34 (9.8%)
Valvular defects	33 (9.5%)
Asthma	30 (8.7%)
Cancer	27 (7.8%)
Stroke history	23 (6.6%)
Thrombosis	22 (6.4%)
Obstructive pulmonary disease	21 (6.1%)
Other thyroid diseases	19 (5.5%)
Hyperthyroidism	9 (2.6%)
Cardiomyopathy	6 (1.7%)
Previous cardiac procedures, n (%)	
Angioplasty	85 (24.6%)
Coronary artery bypass grafting	24 (6.9%)
Implanted cardioverter	18 (5.2%)
Medications, n (%)	
Beta-blocker	292 (84.4%)
Angiotensin-converting enzyme inhibitor	215 (62.1%)
Loop diuretic	161 (46.5%)
Oral anticoagulant	133 (38.4%)
Thiazide diuretic	121 (35.0%)
SGLT2 inhibitor	86 (24.9%)
P2Y12 inhibitor	34 (9.8%)
Sacubitril/valsartan	13 (3.8%)

**Table 2:** Analysis of differences in HRQL measures between the initial and final questionnaires.

	Mean	sd	*p*-Value	95% CI
CHFQ				
Dyspnea	1.41	5.65	<0.001	(0.79; 2.02)
Fatigue	−0.03	8.12	0.95	(−0.95; 0.90)
Emotion	1.01	4.71	<0.001	(0.49; 1.54)
EQ-5D-5L				
EQ VAS	5.02	21.5	<0.001	(2.73; 7.30)

**Table 3:** Multivariate regression analysis for statistically significant subscale scores changes.

	Dyspnoea		Emotional Function		EQVAS	
	Coefficient	*p*-Value	Coefficient	*p*-Value	Coefficient	*p*-Value
Number of telemedical examinations	−0.004	0.078	0.002	0.2601	−0.016	0.094
Number of pharmacotherapy modifications	0.339	0.113	−0.315	0.0862	−1.829	0.025
Sex (female = 1)	1.946	0.003	0.810	0.1503	1.039	0.676
Age	−0.020	0.511	−0.025	0.3447	−0.170	0.147
NYHA class	1.202	0.011	1.534	0.0003	−0.240	0.893
Constant	−0.147	0.951	−0.980	0.6417	22.643	0.015

## Data Availability

All collected data are the property of the Department of Cardiology the University of Rzeszow and will be preserved there.
